# The Endothelial Activation and Stress Index (EASIX) score is an independent prognostic factor in patients with diffuse large B-cell lymphoma

**DOI:** 10.1186/s12885-022-09915-4

**Published:** 2022-07-25

**Authors:** Sungwoo Park, Se-Il Go, Gyeong-Won Lee

**Affiliations:** 1 Division of Hematology and Oncology, Department of Internal Medicine, Gyeongsang National University Hospital, Gyeongsang National University College of Medicine, Jinju, South Korea; 2 Division of Hematology and Oncology, Department of Internal Medicine, Institute of Health Sciences, Gyeongsang National University Changwon Hospital, Gyeongsang National University College of Medicine, Changwon, South Korea

**Keywords:** Lymphoma, Large B-cell, Diffuse, Endothelial Activation and Stress Index, Prognosis, Drug-related side effects and adverse reactions

## Abstract

**Background:**

The endothelial activation and stress index (EASIX) score has been reported to predict overall survival (OS) in hematological cancers. However, it has not been validated as a prognostic marker for diffuse large B-cell lymphoma (DLBCL) to date.

**Methods:**

The records of 265 patients who presented with DLBCL in the Republic of Korea between January 07, 2004, and March 05, 2020 were retrospectively reviewed. For all included patients, EASIX scores were calculated using serum lactate dehydrogenase (LDH) and creatinine levels and the platelet count measured at diagnosis as follows: LDH (U/L) × creatinine (mg/dL)/platelet count (10^9^/L).

**Results:**

The median age of the patients was 64 years. The optimal cutoff value of EASIX according to the receiver operating characteristic analysis for OS was 1.33. All the patients were treated with cyclophosphamide, doxorubicin, vincristine, and prednisone combined with rituximab. The 1-year OS and progression-free survival (PFS) rates were lower in the high-EASIX group than in the low EASIX group (63.8% vs. 84.4%, *p* < 0.001 and 54.0% vs. 79.6%, *p* < 0.001, respectively). A high EASIX was an independent poor prognostic factor for OS and PFS (hazard ratio, 1.606; 95% CI, 1.077–2.395; *p* = 0.020 and hazard ratio, 1.621; 95% CI, 1.066–2.464; *p* = 0.024, respectively).

**Conclusions:**

EASIX is a readily available and cheaply obtainable parameter in clinical studies and shows considerable potential as a new prognostic marker for patients with newly diagnosed DLBCL.

**Supplementary Information:**

The online version contains supplementary material available at 10.1186/s12885-022-09915-4.

## Background

Diffuse large B-cell lymphoma (DLBCL) is the most common type of non-Hodgkin lymphoma (NHL) in the world, accounting for 30–40% of all adult NHLs [1]. With the addition of new drugs to the standard R-CHOP-21 regimen for DLBCL, newer prognostic and predictive factors need to be developed based on the biology of DLBCL [2]. The International Prognostic Index (IPI) remains the most robust prognostic tool for DLBCL and has been used in all clinical trials to date [2]. However, it has limited ability to predict patients who will experience a particularly aggressive disease course, since even the “high-risk” group has a 50% chance of 3-year event-free survival [2].

Angiogenesis plays a critical role in tumor progression. The interplay between the lymphoma microenvironment and angiogenesis has been suggested to contribute to poor clinical outcomes in DLBCL [3–5]. However, the addition of antiangiogenic agents to the R-CHOP regimen failed to demonstrate survival benefits [6, 7]. Clinically relevant biomarkers for angiogenesis are required to identify patients who would benefit from antiangiogenic therapy. Several angiogenesis-related markers, including microvessel density, circulating endothelial cells, and tumor microenvironment, have been reported to be associated with the prognosis of patients with B-cell NHL [8]. The endothelial activation and stress index (EASIX) was recently developed with parameters used to diagnose thrombotic microangiopathy, an endothelial dysfunction disorder [9]. The components of EASIX act as surrogate markers of endothelial dysfunction, and their levels reflect the degree of endothelial dysfunction [9]. A group from Germany and the United States reported that the EASIX is a reliable factor to predict the prognosis of acute graft-versus-host disease after allogeneic stem cell transplantation [9]. They subsequently suggested that EASIX could predict survival outcomes in lower-risk myelodysplastic syndrome, which is not a candidate for allogeneic stem cell transplantation [10]. The prognostic influence of EASIX in allogeneic stem cell transplantation was externally validated in generalized population cohorts [11–13]. EASIX was also recently suggested to serve as a simple, universal prognostic index for survival in COVID-19 patients with and without hematological malignancies [14].

Platelet count, serum creatinine, and lactate dehydrogenase (LDH), which constitute EASIX, are well-known or reported prognostic factors for lymphoma [15–17]. Therefore, we planned this study to determine whether EASIX could also be useful in predicting the survival outcomes of patients with DLBCL.

## Methods

### Data collection

This study retrospectively reviewed the medical records of 265 patients presenting with DLBCL between January 07, 2004 and March 05, 2020. EASIX was calculated using the following formula: LDH (U/L) × creatinine (mg/dL) / platelet count (10^9^/L) [9]. To calculate EASIX, we used these laboratory data which were collected on the nearest day within a week before the beginning of treatment. Staging procedures included clinical examination; computerized tomography (CT) of the chest, abdomen, and pelvis (and neck if indicated); blood count and clinical biochemistry assessments; bone marrow biopsy; and echocardiography. Performance status was assessed using the Eastern Cooperative Oncology Group (ECOG) scale [18]. Bulky disease was defined by the presence of a mediastinal mass exceeding one-third of the maximum intrathoracic diameter or a nodal mass larger than 7.5 cm. Tumor responses were assessed after every three cycles of chemotherapy and at the end of treatment and were classified according to the Lugano classification lymphoma response criteria [19]. PET-CT was performed at the end of chemotherapy to confirm complete response (CR). Toxicity was evaluated using the NCI Common Terminology Criteria for Adverse Events (CTCAE) v5.0 toxicity scale (http://ctep.cancer.gov/protocolDevelopment/electronic applications/docs/ctcaev5.pdf). The exclusion criteria were as follows: a recent history of other malignant diseases except for non-melanoma skin cancer or carcinoma in situ of the cervix, prior radiotherapy or chemotherapy, history of indolent lymphoma, central nervous system involvement at diagnosis, seropositivity for human immunodeficiency virus, pregnancy or breastfeeding, mental illness, or other serious inflammatory conditions. The Institutional Review Board of Gyeongsang National University Hospital approved this retrospective study and waived the requirement for informed consent.

### Statistical analysis

The chi-square method was used for group comparisons of categorical variables. Descriptive statistics were reported as proportions and medians. Overall survival (OS) was calculated from the start of treatment until death from any cause. Progression-free survival (PFS) was measured from the start of treatment to death from any cause, progression during or after treatment, or the last follow-up at which the patient was known to be alive. Survival curves for OS and PFS were plotted using the Kaplan–Meier method, and differences between curves were evaluated using log-rank tests. The optimal cutoff value for EASIX was determined using receiver operating characteristic curve analysis to predict OS and PFS. The baseline characteristics and clinical outcomes were compared between the two groups dichotomized according to the cutoff value. Univariate analysis with the log-rank test was used to evaluate prognostic factors for PFS and OS. Multivariate analysis was performed using the Cox proportional hazard regression model. The following factors were assessed for their influence on survival: sex, presence of B symptoms, presence of bulky disease, bone marrow involvement, IPI (determined by age, ECOG performance status, tumor stage, LDH, and extranodal involvement) and its variants, and EASIX. Variables with *p*-values less than 0.1 in the univariate analysis were entered into the multivariate model. Factors with *p-*values < 0.05 were considered significant. The variance inflation factor (VIF) was calculated to evaluate multicollinearity between variables, with tolerance < 0.1 and VIF > 10 considered indicative of multicollinearity. The Harrell’s c-index was calculated to evaluate and compare the predictive performance of models. All analyses were performed using the Stata software version 16.1 (Stata Corp, College Station, TX, USA).

## Results

### Patient characteristics

The median patient age was 64 years (range, 21–88 years), and 148 patients (55.8%) were male. A total of 47 patients (17.7%) experienced B symptoms. One hundred fifty-two patients (57.4%) presented with Ann Arbor stages III-IV, and 124 patients (46.7%) presented with high–intermediate- or high-risk IPI. Forty-six patients (17.4%) showed germinal center B (GCB) cell type, and in 92 (34.7%) cases, the cell of origin could not be identified. Fifty-one patients (19.2%) showed bulky disease. All the patients were treated with cyclophosphamide, doxorubicin, vincristine, and prednisone combined with rituximab. Other patient characteristics are summarized in Table 1.

**Table 1:** Patient demographics and characteristics according to the EASIX score

Factor	Total (*n* = 265)	Low EASIX (*n* = 172)	High EASIX (*n* = 93)	*P*
Sex				0.428
Male	148 (55.8)	93 (54.1)	55 (59.1)	
Female	117 (44.2)	79 (45.9)	38 (40.9)	
Age, years				< 0.001
≤ 60	102 (38.5)	83 (48.3)	24 (25.8)	
> 60	163 (61.5)	89 (51.7)	69 (74.2)	
ECOG PS				< 0.001
0–1	189 (71.3)	140 (81.4)	49 (52.7)	
2–3	76 (28.7)	32 (18.6)	44 (47.3)	
Symptom stage				< 0.001
A	218 (82.3)	153 (89.0)	65 (69.9)	
B	47 (17.7)	19 (11.1)	28 (30.1)	
Ann Arbor Stage				< 0.001
I-II	113 (42.6)	90 (52.3)	23 (24.7)	
III-IV	152 (57.4)	82 (47.7)	70 (75.3)	
IPI				< 0.001
Low	86 (32.5)	78 (45.4)	8 (8.6)	
Low-intermediate	55 (20.8)	42 (24.4)	13 (14.0)	
High-intermediate	56 (21.1)	26 (15.1)	30 (32.3)	
High	68 (25.6)	26 (15.1)	42 (45.2)	
Revised IPI				< 0.001
Very good	36 (13.6)	35 (20.4)	1 (1.1)	
Good	105 (39.6)	85 (49.4)	20 (21.5)	
Poor	124 (46.8)	52 (30.2)	72 (77.4)	
NCCN-IPI				< 0.001
Low	25 (9.4)	22 (12.8)	3 (3.2)	
Low-intermediate	94 (35.5)	82 (47.7)	12 (12.9)	
High-intermediate	89 (33.6)	52 (30.2)	37 (39.8)	
High	57 (21.5)	16 (9.3)	41 (44.1)	
Bone marrow involvement				< 0.001
Yes	231 (87.2)	13 (7.6)	21 (22.6)	
No	34 (12.8)	159 (92.4)	72 (77.4)	
Number of extranodal sites of involvement				< 0.001
< 2	182 (68.7)	132 (76.7)	50 (53.8)	
≥ 2	83 (31.3)	40 (23.3)	43 (46.2)	
Tumor lysis syndrome				< 0.001
Yes	12 (4.5)	1 (0.6)	11 (11.8)	
No	253 (95.5)	171 (99.4)	82 (88.2)	
Renal involvement of lymphoma				0.040
Yes	14 (5.3)	5 (2.9)	9 (9.7)	
No	251 (94.7)	167 (97.1)	84 (90.3)	
Chronic kidney disease				> 0.998
Yes	9 (3.4)	6 (3.5)	3 (3.2)	
No	256 (96.6)	166 (96.5)	90 (96.8)	
Bulky disease				0.769
Yes	214 (80.8)	34 (19.8)	17 (18.3)	
No	51 (19.2)	138 (80.2)	76 (81.7)	
Lactate dehydrogenase				< 0.001
Elevated	108 (40.8)	72 (41.9)	85 (91.4)	
Normal	157 (59.2)	100 (58.1)	8 (8.6)	
Cell of origin				0.113
GCB	46 (17.4)	26 (15.1)	20 (21.5)	
Non-GCB	127 (47.9)	79 (45.9)	48 (51.6)	
Not available	92 (34.7)	67 (39.0)	25 (26.9)	

Data are presented as the number of patients (%).

*EASIX* Endothelial activation and stress index, *ECOG PS* Eastern Cooperative Oncology Group performance status, *IPI* International Prognostic Index, *NCCN-IPI* National Comprehensive Cancer Network International Prognostic Index, *GCB* Germinal center.

### Treatment response

The CR, partial response (PR), no response or stable disease (NR/SD), and progressive disease (PD) rates were 80.8, 14.0, 1.7, and 3.5% in the low EASIX group and 61.3, 22.6, 3.2, and 12.9% in the high-EASIX group, respectively (*p* < 0.001). In a subgroup analysis of patients who did not early discontinue treatment, except for the progression of lymphoma, the CR rates were 88.7 and 79.1% in the low- and high-EASIX groups, respectively (*p* = 0.060).

### Survival outcome

**Fig. 1:**
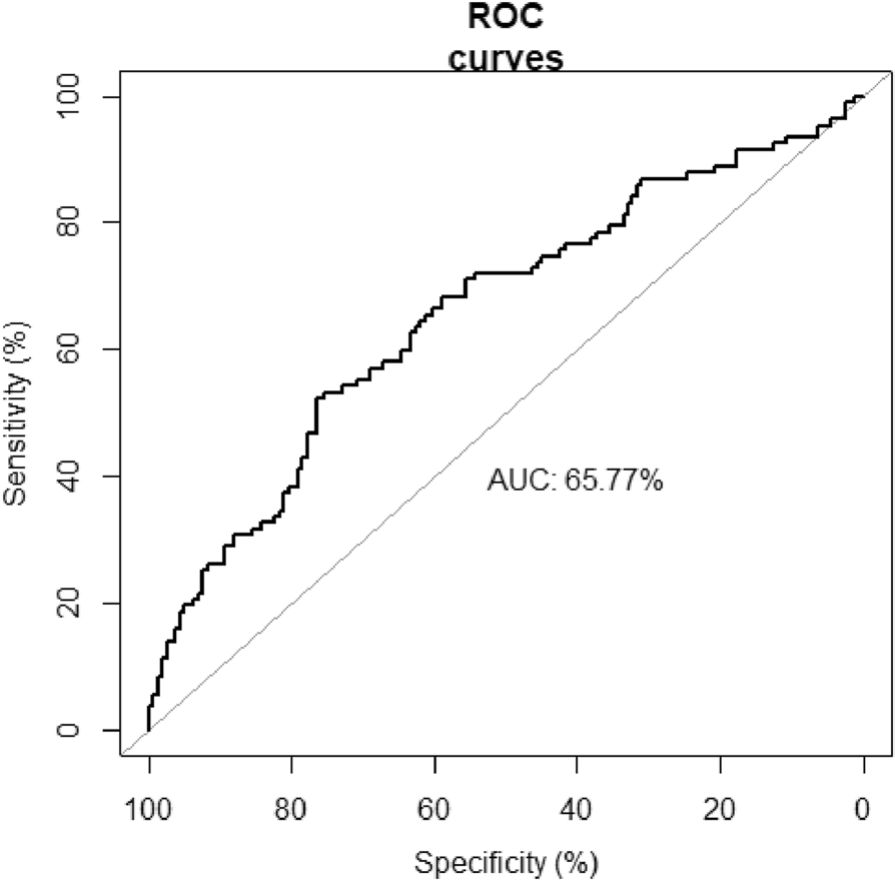
ROC curves for comparison ROC, receiver operating characteristic; AUC, area under the curve.

**Fig. 2:**
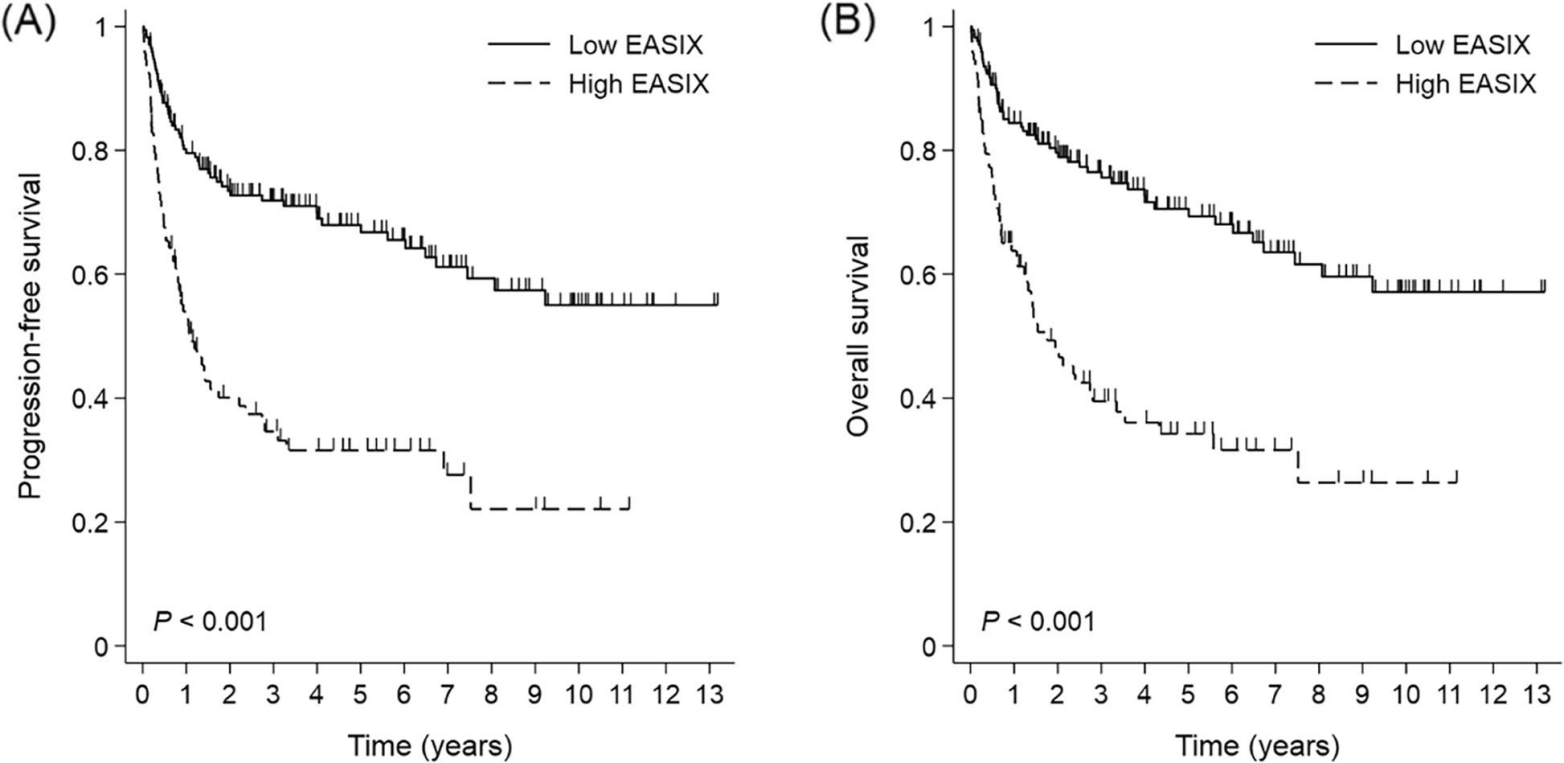
Kaplan–Meier survival curves for progression-free survival (**A**) and overall survival (**B**) according to the endothelial activation and stress index (EASIX) scores

At a median follow-up period of 57 months, 118 patients (44.5%) showed disease relapse and 107 patients (40.4%) had died. The 5-year PFS and OS rates were 55.1 and 57.9%, respectively. On the basis of the cutoff EASIX score of 1.33 (Fig. 1), 93 and 172 patients were categorized as having high and low EASIX, respectively. The 5-year PFS (67.9% vs. 31.6%) and 5-year OS (70.6% vs. 34.3%) rates differed significantly between the low- and high-EASIX groups (*p* < 0.001, Fig. 2).

### Factors affecting overall survival and progression-free survival

**Fig. 3:**
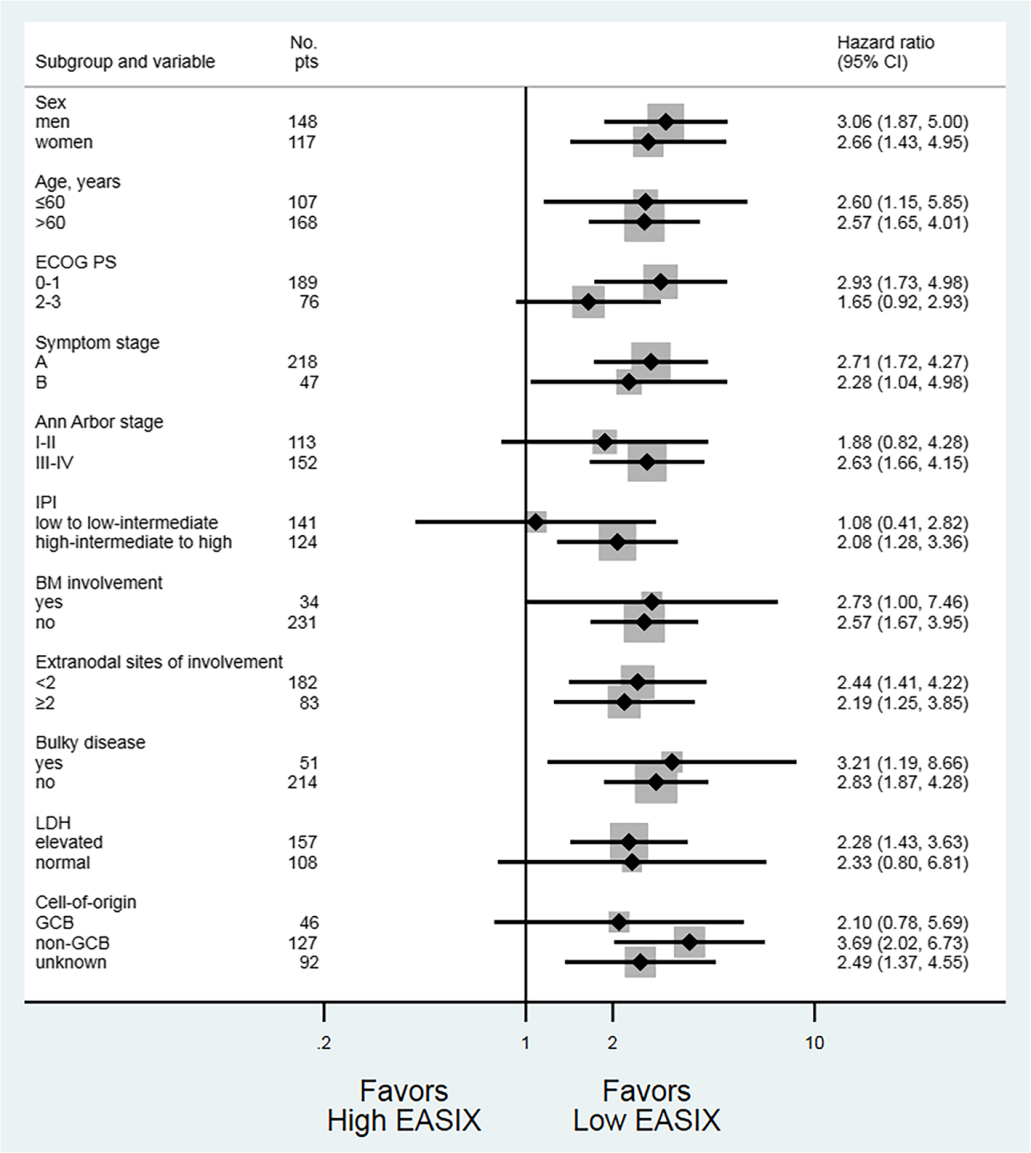
Forest plot for overall survival by subgroup. EASIX, endothelial activation and stress index; ECOG PS, Eastern Cooperative Oncology Group performance status; IPI, International Prognostic Index; BM, bone marrow; LDH, lactate dehydrogenase; GCB, germinal center B-cell.

The symptom stage (B vs. A), IPI (high–intermediate to high vs. low-to-low-intermediate), bone marrow involvement (yes vs. no), tumor lysis syndrome (yes vs. no), chronic kidney disease (yes vs. no), and EASIX score (high vs. low) were significantly associated with OS and PFS in a univariate analysis. In multivariate analysis, IPI (high–intermediate to high vs. low to low-intermediate) and EASIX score (high vs. low) were significant prognostic factors (Table 2). There was no multicollinearity among the factors because the VIF value was less than 1.5. When the revised- or NCCN-IPI instead of original IPI was included in the Cox regression model for OS, similar results were observed (see Additional file 1). The Harrell’s c-indices were 0.73, 0.74, and 0.73 in the Cox regression model including IPI, revised-IPI, or NCCN-IPI, respectively. The OS results favored the low EASIX group in most subgroups based on disease characteristics at baseline (Fig. 3).

**Table 2:** Univariate and multivariate Cox regression analyses for PFS and OS

	Progression-free survival			
	Univariate		Multivariate	
	HR (95% CI)	*P*	HR (95% CI)	*P*
Sex (men vs. women)	1.245 (0.861–1.801)	0.244		
Symptom stage (B vs. A)	2.270 (1.517–3.398)	< 0.001	1.480 (0.974–2.250)	0.066
IPI (HI to high vs. low to LI)	5.002 (3.309–7.562)	< 0.001	3.662 (2.326–5.765)	< 0.001
BM involvement (yes vs. no)	2.664 (1.704–4.162)	< 0.001	1.361 (0.844–2.193)	0.206
Tumor lysis syndrome (yes vs. no)	3.228 (1.631–6.388)	0.001	1.620 (0.802–3.270)	0.178
Renal involvement (yes vs. no)	2.659 (1.382–5.116)	0.003	1.109 (0.563–2.186)	0.765
Chronic kidney disease (yes vs. no)	0.688 (0.219–2.164)	0.522		
Bulky disease (yes vs. no)	0.777 (0.470–1.284)	0.325		
EASIX (high vs. low)	2.879 (1.998–4.148)	< 0.001	1.551 (1.034–2.326)	0.034
	Overall survival			
	Univariate		Multivariate	
	HR (95% CI)	*P*	HR (95% CI)	*P*
Sex (men vs. women)	1.297 (0.878–1.916)	0.191		
Symptom stage (B vs. A)	2.208 (1.447–3.371)	< 0.001	1.467 (0.947–2.274)	0.086
IPI (HI to high vs. low to LI)	5.104 (3.300–7.894)	< 0.001	3.763 (2.338–6.055)	< 0.001
BM involvement (yes vs. no)	2.484 (1.547–3.988)	< 0.001	1.278 (0.771–2.118)	0.342
Tumor lysis syndrome (yes vs. no)	3.139 (1.522–6.472)	0.002	1.574 (0.747–3.317)	0.233
Renal involvement (yes vs. no)	3.070 (1.587–5.939)	0.001	1.322 (0.667–2.618)	0.423
Chronic kidney disease (yes vs. no)	0.440 (0.109–1.784)	0.250		
Bulky disease (yes vs. no)	0.762 (0.448–1.298)	0.317		
EASIX (high vs. low)	2.898 (1.975–4.253)	< 0.001	1.567 (1.024–2.398)	0.038

*HR* Hazard ratio, *CI* Confidence interval, *IPI* International Prognostic Index, *HI* High-intermediate, *LI* Low-intermediate, *BM* Bone marrow, *EASIX* Endothelial activation and stress index

### Toxicity

Table 3 lists the incidences of the toxicities according to the NCI-CTC toxicity criteria. Hematologic toxicity grade ≥ 3 was reported in 210 patients (79.2%) at least once in the course of chemotherapy. Non-hematologic toxicity grade ≥ 3 was reported in 101 patients (38.1%). The low- and high-EASIX groups showed significant differences in terms of the incidence of hematologic toxicity grade ≥ 3, non-hematologic toxicity grade ≥ 3, dose reduction, and early treatment discontinuation. Treatment-related mortality did not exhibit a significant difference between the groups.

**Table 3:** Treatment-related toxicities

	Total (*n* = 265)	Low EASIX (*n* = 172)	High EASIX (*n* = 93)	*P*
Hematologic toxicity, grade ≥ 3				
Anemia	53 (20)	21 (12.2)	32 (34.4)	< 0.001
Thrombocytopenia	75 (28.3)	25 (14.5)	50 (53.8)	< 0.001
Neutropenia	206 (77.7)	124 (72.1)	82 (88.2)	0.003
Febrile neutropenia	76 (28.7)	32 (18.6)	44 (47.3)	< 0.001
Non-hematologic toxicity, grade ≥ 3	101 (38.1)	55 (32.0)	46 (49.5)	0.005
Treatment-related mortality	22 (8.3)	11 (6.4)	11 (11.8)	0.126
Dose reduction	125 (47.2)	69 (40.1)	56 (60.2)	0.002
Early treatment discontinuation	47 (17.7)	21 (12.2)	26 (28.0)	0.001

Data are presented as the number of patients (%)

*EASIX* Endothelial activation and stress index, *RDI* Relative dose intensity

## Discussion

The EASIX score is easy to calculate, and any hematology center now has the opportunity to validate EASIX scores in its patient cohort [13]. Therefore, the main strength of our approach is the use of standard parameters that are readily available in routine clinical practice [9]. The EASIX score is a simple score based on three laboratory parameters used worldwide [13]. The creatinine and LDH levels and platelet counts used for determining EASIX are connected to endothelial pathology [20, 21]. High creatinine levels connect endothelial pathology to renal function since endothelial pathomechanisms influence conditions such as acute renal failure, glomerulonephritis, diabetic nephropathy, and transplant glomerulonephropathy [22]. LDH is also involved because endothelial activation leads to the release of LDH from endothelial cells, as well as to a higher turnover of circulating cells, such as thrombocytes and leukocytes, resulting in elevated LDH serum levels [23, 24]. Low platelet counts result from endothelial damage and complement activation in many diseases, such as thrombotic microangiopathies [24], chronic graft-versus-host disease [25], adult respiratory distress syndrome [26], and other critical conditions.

For NHLs, the importance of angiogenesis in human lymphoma has been well recognized over the last 20 years [27]. Among the different types of B-cell NHLs, angiogenesis may be prominent in aggressive rather than indolent subtypes [27]. Endothelial mechanisms play a major role in angiogenesis [28]. Routine laboratory parameters associated with EASIX scores are associated with the prognosis of lymphoma, and serum LDH levels are commonly elevated in patients with lymphoproliferative disorders. In patients with NHL, LDH levels are of prognostic importance and can thus be used to monitor treatment response and recurrence, if any [15]. Pretreatment renal impairment is an independent prognostic marker of inferior OS in DLBCL [16], and thrombocytopenia at diagnosis has been reported to be an independent prognostic factor for survival in patients with PTCL [17]. Therefore, we assumed that EASIX scores might be important for prognostic stratification of lymphoma as an endothelial dysfunction-related marker independent of other prognostic factors.

In contrast, a recent classification study for lymphoma microenvironment (LME) of 4655 DLBCL patients treated with R-CHOP [29] reported that mesenchymal (MS) subtype indicating a higher proportion of signatures from vascular endothelial cells had a more favorable prognosis than inflammatory (IN) and depleted (DP) subtypes and similarly favorable prognosis with germinal center-like (GC) subtype. The reason why the prognosis of MS subtype and high EASIX group was different is unclear. A possible explanation is that these two indices have additional confounding components for prognosis besides endothelial activation and dysfunction. For example, the proportion of poor prognostic markers such as activated B-cell subtype and co-occurrence of *MYD88* and *CD79B* mutations were lower in MS subtype than in IN and DP subtypes. EASIX may also be affected by many clinical conditions such as infection, tumor lysis syndrome, bone marrow involvement of lymphoma, and drugs. Therefore, further study using additional biomarker for endothelial cell proliferation is needed to confirm the prognostic impact of endothelial activation and dysfunction and it is necessary to evaluate the prognostic value of EASIX according to the LME subtype.

EASIX-based assessments may show some limitations. First, as described above, the parameters included in EASIX are non-specific because of many confounding factors, such as transfusions, infections, corticosteroids, and other conditions including tumor lysis syndrome, chronic kidney disease, and renal involvement of lymphoma. Only nine of 265 patients (3.4%) preemptively used corticosteroids before the beginning of R-CHOP. In multivariate analysis including several confounding factors described above, the prognostic impact of EASIX was maintained. However, the confounding effects of these factors on EASIX cannot be neglected in the evaluation for clinical implication of endothelial dysfunction in DLBCL. Second, since the introduction of next-generation sequencing methods, genetic research has been actively conducted on lymphoma. However, the genetic profile of the patient group was not analyzed in this study. We could not determine whether EASIX could overcome the effects of genes on prognosis. Finally, the distribution of patients in the low-and high-EASIX groups was uneven, except for sex and bulky disease. However, we attempted to minimize the error caused by this factor by correcting the confusion variable, which is a prognostic factor that can operate independent of IPI. In addition, because we identified the association of EASIX scores with treatment-related toxicity and PFS in lymphoma patients, it is possible to more clearly define patient groups requiring targeted treatments that have relatively fewer side effects but are more expensive than traditional chemotherapy.

## Conclusions

This study evaluated the prognostic value of EASIX in patients with newly diagnosed DLBCL. Patients with high EASIX values at diagnosis had significantly inferior survival outcomes and showed more severe toxicity than those with low EASIX values. Therefore, EASIX may be a potential predictor of survival outcome in patients with newly diagnosed DLBCL and its validity should be confirmed through the external validation cohort.

## Supplementary Information

**Additional file 1.**

## Data Availability

The datasets used and/or analyzed during the current study are available from the corresponding author on reasonable request.

## Abbreviations

CR: Complete response
CT: Computerized tomography
CTCAE: Common Terminology Criteria for Adverse Events
DLBCL: Diffuse large B-cell lymphoma
EASIX: Endothelial activation and stress index
ECOG: Eastern Cooperative Oncology Group
IPI: International Prognostic Index
LDH: Lactate dehydrogenase
NHL: Non-Hodgkin lymphoma
OS: Overall survival
PD: Progressive disease
PFS: Progression-free survival
PR: Partial response
VIF: Variance inflation factor

## References

[1] Siegel RL, Miller KD, Jemal A (2017). Cancer statistics, 2017. CA Cancer J Clin.

[2] Vaidya R, Witzig TE (2014). Prognostic factors for diffuse large B-cell lymphoma in the R(X) CHOP era. Ann Oncol.

[3] Lenz G, Wright G, Dave SS, Xiao W, Powell J, Zhao H (2008). Stromal Gene Signatures in Large-B-Cell Lymphomas. N Engl J Med.

[4] Carbone A, Gloghini A (2013). The microenvironment of AIDS-related diffuse large B-cell lymphoma provides insight into the pathophysiology and indicates possible therapeutic strategies. Blood..

[5] Suhasini AN, Wang L, Holder KN, Lin AP, Bhatnagar H, Kim SW (2016). A phosphodiesterase 4B-dependent interplay between tumor cells and the microenvironment regulates angiogenesis in B-cell lymphoma. Leukemia..

[6] Seymour JF, Pfreundschuh M, Trněný M, Sehn LH, Catalano J, Csinady E (2014). R-CHOP with or without bevacizumab in patients with previously untreated diffuse large B-cell lymphoma: Final MAIN study outcomes. Haematologica..

[7] Pasvolsky O, Rozental A, Raanani P, Gafter-Gvili A, Gurion R (2021). R-CHOP compared to R-CHOP + X for newly diagnosed diffuse large B-cell lymphoma: a systematic review and meta-analysis. Acta Oncol (Madr).

[8] Jiang L, Li N (2020). B-cell non-Hodgkin lymphoma: Importance of angiogenesis and antiangiogenic therapy. Angiogenesis..

[9] Luft T, Benner A, Jodele S, Dandoy CE, Storb R, Gooley T (2017). EASIX in patients with acute graft-versus-host disease: a retrospective cohort analysis. Lancet Haematol.

[10] Merz A, Germing U, Kobbe G, Kaivers J, Jauch A, Radujkovic A (2019). EASIX for prediction of survival in lower-risk myelodysplastic syndromes. Blood Cancer J.

[11] Shouval R, Fein JA, Shouval A, Danylesko I, Shem-Tov N, Zlotnik M (2019). External validation and comparison of multiple prognostic scores in allogeneic hematopoietic stem cell transplantation. Blood Adv.

[12] Varma A, Rondon G, Srour SA, Chen J, Ledesma C, Champlin RE (2020). Endothelial Activation and Stress Index (EASIX) at Admission Predicts Fluid Overload in Recipients of Allogeneic Stem Cell Transplantation. Biol Blood Marrow Transplant.

[13] Luft T, Benner A, Terzer T, Jodele S, Dandoy CE, Storb R (2020). EASIX and mortality after allogeneic stem cell transplantation. Bone Marrow Transplant.

[14] Kalicińska E, Biernat M, Rybka J, Zińczuk A, Janocha-litwin J, Rosiek-biegus M (2021). Endothelial activation and stress index (Easix) as an early predictor for mortality and overall survival in hematological and non-hematological patients with covid-19: Multicenter cohort study. J Clin Med.

[15] Yadav C, Ahmad A, D’Souza B, Agarwal A, Nandini M, Ashok Prabhu K (2016). Serum Lactate Dehydrogenase in Non-Hodgkin’s Lymphoma: A Prognostic Indicator. Indian J Clin Biochem.

[16] Hong J, Lee S, Chun G, Jung JY, Park J, Ahn JY (2016). Baseline renal function as a prognostic indicator in patients with newly diagnosed diffuse large B-cell lymphoma. Blood Res.

[17] Choi M, Lee JO, Jung J, Lee JY, Lee E, Lee H (2019). Prognostic value of platelet count in patients with peripheral T cell lymphoma. Acta Haematol.

[18] Oken M, Creech R, Tormey D, Horton J, Davis T, McFadden E (1982). Toxicity and response criteria of the Eastern Cooperative Oncology Group. Am J Clin Oncol.

[19] Cheson BD, Ansell S, Schwartz L, Gordon LI, Advani R, Jacene HA (2016). Refinement of the Lugano Classification lymphoma response criteria in the era of immunomodulatory therapy. Blood..

[20] Ho VT, Cutler C, Carter S, Martin P, Adams R, Horowitz M (2005). Blood and Marrow Transplant Clinical Trials Network Toxicity Committee consensus summary: Thrombotic microangiopathy after hematopoietic stem cell transplantation. Biol Blood Marrow Transplant..

[21] Ruutu T, Barosi G, Benjamin RJ, Clark RE, George JN, Gratwohl A (2007). Diagnostic criteria for hematopoietic stem cell transplant-associated microangiopathy: Results of a consensus process by an International Working Group. Haematologica..

[22] Hanf W, Bonder CS, Coates PTH (2014). Transplant glomerulopathy: The interaction of HLA antibodies and endothelium. J Immunol Res.

[23] Chopra J, Joist JH, Webster RO (1987). Loss of 51Chromium, lactate dehydrogenase, and 111Indium as indicators of endothelial cell injury. Lab Investig.

[24] Coppo P, Schwarzinger M, Buffet M, Wynckel A, Clabault K, Presne C (2010). Predictive features of severe acquired ADAMTS13 deficiency in idiopathic thrombotic microangiopathies: The French TMA reference center experience. PLoS One.

[25] Ayuk F, Veit R, Zabelina T, Bussmann L, Christopeit M, Alchalby H (2015). Prognostic factors for survival of patients with newly diagnosed chronic GVHD according to NIH criteria. Ann Hematol.

[26] Wei Y, Wang Z, Su L, Chen F, Tejera P, Bajwa EK (2015). Platelet count mediates the contribution of a genetic variant in LRRC16A to ARDS risk. Chest..

[27] Ribatti D, Nico B, Ranieri G, Specchia G, Vacca A (2013). The role of angiogenesis in human non-Hodgkin lymphomas. Neoplasia (United States).

[28] Ungvari Z, Tarantini S, Kiss T, Wren JD, Cory B, Griffin CT (2019). Ageing Vasculature. Nat Rev Cardiol.

[29] Kotlov N, Bagaev A, Revuelta MV, Phillip JM, Cacciapuoti MT, Antysheva Z (2021). Clinical and biological subtypes of b-cell lymphoma revealed by microenvironmental signatures. Cancer Discov.

