# Supplementary material for: The Endothelial Activation and Stress Index (EASIX) score is an independent prognostic factor in patients with diffuse large B-cell lymphoma
Source: BMC Cancer. 2022 Jul 25;22:816. doi: 10.1186/s12885-022-09915-4 (PMC9312320; doi:10.1186/s12885-022-09915-4)
Supplement: Supplementary file 1 — Additional file 1. [file 12885_2022_9915_MOESM1_ESM.docx]

**Table S1.** Cox regression analyses for overall survival

|  | Overall survival | | | |  |  |
| --- | --- | --- | --- | --- | --- | --- |
|  | Univariate | | Multivariate (excluding NCCN-IPI) | | Multivariate (excluding R-IPI) | |
|  | HR (95% CI) | *P* | HR (95% CI) | *P* | HR (95% CI) | *P* |
| Sex (men vs. women) | 1.297 (0.878-1.916) | 0.191 |  |  |  |  |
| Symptom stage (B vs. A) | 2.208 (1.447-3.371) | <0.001 | 1.457 (0.941-2.257) | 0.086 | 1.410 (0.909-2.188) | 0.125 |
| Revised-IPI (poor vs. good vs. very good) | 4.164 (2.835-6.117) | <0.001 | 3.332 (2.212-5.018) | <0.001 | Not included | |
| NCCN-IPI (HI to high vs. low to LI) | 6.149 (3.726-10.149) | <0.001 | Not included | | 4.577 (2.690-7.788) | <0.001 |
| BM involvement (yes vs. no) | 2.484 (1.547-3.988) | <0.001 | 1.259 (0.763-2.079) | 0.368 | 1.389 (0.839-2.298) | 0.201 |
| Tumor lysis syndrome (yes vs. no) | 3.139 (1.522-6.472) | 0.002 | 1.568 (0.744-3.301) | 0.237 | 1.370 (0.648-2.894) | 0.410 |
| Renal involvement (yes vs. no) | 3.070 (1.587-5.939) | 0.001 | 1.316 (0.666-2.601) | 0.429 | 1.447 (0.731-2.867) | 0.289 |
| Chronic kidney disease (yes vs. no) | 0.440 (0.109-1.784) | 0.250 |  |  |  |  |
| Bulky disease (yes vs. no) | 0.762 (0.448-1.298) | 0.317 |  |  |  |  |
| EASIX (high vs. low) | 2.898 (1.975-4.253) | <0.001 | 1.474 (0.968-2.244) | 0.070 | 1.548 (1.015-2.362) | 0.043 |

HR, hazard ratio; CI, confidence interval; IPI, International Prognostic Index; HI, high-intermediate; LI, low-intermediate; NCCN, national comprehensive cancer network; BM, bone marrow; EASIX, endothelial activation and stress index
